# Association of genetic risk and physical activity with incident type 2 diabetes

**DOI:** 10.1210/clinem/dgag083

**Published:** 2026-02-27

**Authors:** Xuan Zhou, Germán D Carrasquilla, Malene R Christiansen, Roelof A J Smit, Lars Ängquist, Torben Hansen, Ruth J F Loos, Niels Grarup, Allan Linneberg, Tuomas O Kilpeläinen, Jordi Merino

**Affiliations:** Novo Nordisk Foundation Center for Basic Metabolic Research, University of Copenhagen, Copenhagen 2200, Denmark; Novo Nordisk Foundation Center for Basic Metabolic Research, University of Copenhagen, Copenhagen 2200, Denmark; Novo Nordisk Foundation Center for Basic Metabolic Research, University of Copenhagen, Copenhagen 2200, Denmark; Novo Nordisk Foundation Center for Basic Metabolic Research, University of Copenhagen, Copenhagen 2200, Denmark; Charles Bronfman Institute for Personalized Medicine, Icahn School of Medicine at Mount Sinai, NewYork, NY 10029, USA; Novo Nordisk Foundation Center for Basic Metabolic Research, University of Copenhagen, Copenhagen 2200, Denmark; Novo Nordisk Foundation Center for Basic Metabolic Research, University of Copenhagen, Copenhagen 2200, Denmark; Novo Nordisk Foundation Center for Basic Metabolic Research, University of Copenhagen, Copenhagen 2200, Denmark; Charles Bronfman Institute for Personalized Medicine, Icahn School of Medicine at Mount Sinai, NewYork, NY 10029, USA; Novo Nordisk Foundation Center for Basic Metabolic Research, University of Copenhagen, Copenhagen 2200, Denmark; Center for Clinical Research And Prevention, Copenhagen University Hospital—Bispebjerg and Frederiksberg, the Capital Region, Copenhagen 2000, Denmark; Department of Clinical Medicine, Faculty Of Health And Medical Sciences, University of Copenhagen, Copenhagen 2200, Denmark; Novo Nordisk Foundation Center for Basic Metabolic Research, University of Copenhagen, Copenhagen 2200, Denmark; Novo Nordisk Foundation Center for Basic Metabolic Research, University of Copenhagen, Copenhagen 2200, Denmark; Diabetes Unit And Center For Genomic Medicine, Massachusetts General Hospital, Boston, MA 02114, USA

**Keywords:** type 2 diabetes, physical activity, genetics, gene–environment interaction, wearables

## Abstract

**Objective:**

The aim of this study was to assess whether daily step counts and genetic risk interact to influence the risk of developing type 2 diabetes.

**Research Design and Methods:**

We analyzed data from 9501 participants in the *All of Us* Research Program with both genetic and wearable device–derived physical activity data and without diabetes at baseline and a median age of 56 years (42–66). Physical activity was quantified using daily step counts. Genetic risk was assessed using a global polygenic score. Incident type 2 diabetes was identified using electronic health record–linked diagnostic codes. Multivariable Cox proportional hazards models estimated hazard ratios (HRs) for type 2 diabetes across genetic risk and physical activity levels. We tested for additive interaction using the relative excess risk due to interaction (RERI). In secondary analyses, we used physical-activity intensity measures using wearable-derived and self-reported intensity levels.

**Results:**

Type 2 diabetes incidence rates ranged from 4.1 per 1000 person-years (95% CI, 2.5-5.7) in individuals with high physical activity and low genetic risk to 18.4 (95% CI, 15.2-21.6) in those with low physical activity and high genetic risk (HR, 6.2 (95% CI: 3.97, 9.6)). A significant additive interaction was observed (RERI, 0.20; 95% CI, 0.04-0.36; *P* = .007), with 15% (95% CI, 2-27) of excess risk attributed to the interaction. Similar interaction patterns were found using device-based intensity metrics and self-reported physical activity measures.

**Conclusion:**

These findings provide evidence of additive interactions between genetic risk and physical activity, underscoring the potential value of integrating genomic and device-derived data to identify individuals who would more likely benefit from increasing physical activity.

The risk of developing type 2 diabetes varies widely across individuals as a result of both genetic susceptibility and non-genetic influences (1, 2), commonly grouped under the term environmental factors, which encompass lifestyle factors, socioeconomic context, environmental exposures, and access to health care. This variability underpins the significance of gene–environment interaction, wherein an individual's genetic profile, together with its downstream molecular effectors, interacts with environmental exposures to uniquely modulate disease risk (3-6). While gene–environment interactions are pervasive in nature (7-9), identifying such interactions for complex human traits and diseases remains challenging (1), with often inconsistent findings reported in the literature (10-13). Some population-based studies have shown that adherence to a healthy lifestyle or higher levels of physical activity can interact with genetic risk for obesity, type 2 diabetes, and cardiovascular disease (12, 13), whereas others have found little or no evidence of such interactions (10, 11). These discrepancies underscore important gaps in our understanding of disease heterogeneity and limit the translation of gene–environment research into prevention strategies. A major contributing factor is the limited precision and depth of behavioral exposure assessment in many studies, particularly when relying on self-reported single time-point measures (14, 15).

Step count provides a simple and interpretable metric of daily ambulatory activity, offering advantages over self-reported physical activity measures or less intuitive accelerometer-derived intensity classifications (16). Prior studies have shown that higher step counts are associated with lower type 2 diabetes risk (17, 18), and that accelerometer-measured intensity-specific physical activity may attenuate type 2 diabetes genetic risk (19). However, previous studies of gene–physical activity interactions have generally relied on single time-point physical activity assessments and have not accounted for the dynamic nature of physical activity over time. Moreover, no prior study has directly investigated how daily step counts interact with genetic risk on the development of type 2 diabetes.

In this study, we analyzed longitudinal data from a population-based cohort in the United States, comprising 9501 adults with objectively measured physical activity over time, to test the hypothesis that physical activity, measured via daily step count, interacts with genetic risk in the development of type 2 diabetes. We further evaluated whether similar patterns were observed using additional activity measures, including device-based intensity levels and self-reported physical activity.

## Methods

### Study design and population

**Figure 1:**
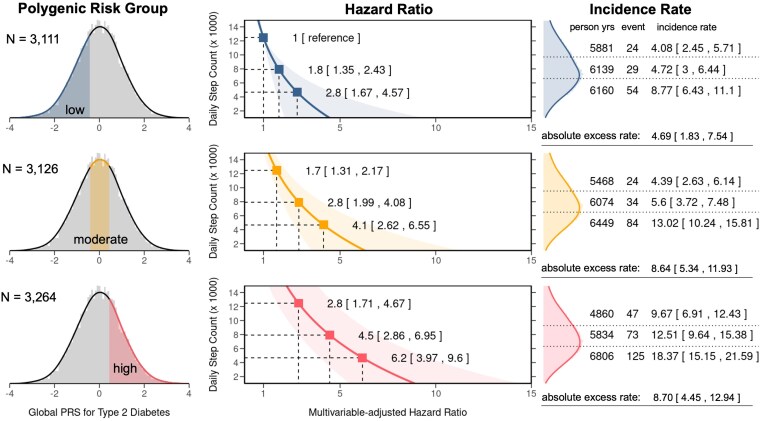
Flow chart of study participants. Among the 14 200 participants from the All of Us cohort with linked Fitbit data, electronic health records, and genetic data at the time of our analysis, 9501 met the criteria for valid physical activity data at baseline and were at least 18 years old during the monitoring period. *EUR* European, *AFR* African, *EAS* East Asian, *SAS* South Asian.

We used data from participants in the *All of Us* Research Program, the design and implementation of which have been described previously (20). This study used data from the Curated Data Repository (CDR) version 8 for the Controlled Tier (C2024Q3R4), including participants enrolled between May 6, 2018, and October 1, 2023. The dataset comprised 633 547 participants with varying availability of physical measurements and vital signs, survey responses, electronic health records (EHR), Fitbit data, and genetic information. The median age of the study population was 52 years (interquartile range [IQR] 37-65), the median body mass index (BMI) was 28.4 kg/m^2^ (IQR 24.4-33.6), and most participants were women (63%) and of European genetic ancestry (68%; Table S1 (21)). Analyses were restricted to participants who owned a Fitbit device and consented to sharing Fitbit, EHR, and genetic data (Fig. 1). Participants who wore a Fitbit for fewer than six months were excluded. Only the authorized authors who completed *All of Us* Research Program Responsible Conduct of Research Training accessed the deidentified data from the Researcher Workbench (a secured cloud-based platform). Since the authors were not directly involved with the participants, institutional review board review was exempted in compliance with *All of Us* Research Program policy. We also used data from Inter99 (22), a population-based pre-randomized lifestyle intervention study aiming to prevent ischemic heart disease and type 2 diabetes, for secondary analyses. Baseline assessments were conducted from March 1999 until January 2001 and included 6784 participants. Follow-up was conducted after 1, 3, 5, 10, and 20 years for a health examination, completion of questionnaires, and risk assessment.

### Physical activity assessment

Activity tracking data for this study came from the Bring Your Own Device program that allowed individuals who already owned a tracking device (Fitbit, Inc) to consent to link their activity data with other data in the *All of Us* cohort. By registering their personal device on the patient portal, participants could share all activity data collected since the creation of their personal device account. We used the mean daily steps calculated on a monthly basis for each participant. Consistent with prior physical activity data curation approaches (17), days with less than 10 hours of wear time, less than 100 steps, or greater than 45 000 steps or for which the participant was younger than 18 years were removed. In addition, we removed months with fewer than 15 valid days of monitoring. To capture additional dimensions of physical activity, we derived device-based measures of intensity, including daily lightly active minutes, moderate active minutes, and very active minutes. We also analyzed self-reported physical activity data from the Inter99 study, assessed via validated questionnaires covering work and leisure-time activity (23), at baseline and at 1-, 3-, and 5-year follow-ups. Reported activity levels were converted into minutes per week by summing responses based on a five-day working week.

### Type 2 diabetes polygenic scores

Detailed descriptions of genotyping, imputation, and quality control in the All of Us Research Program have been published previously (24). Briefly, genotyping was performed at three Genome Centers using a standardized protocol with the Illumina Global Diversity Array to minimize batch effects. Rigorous sample- and batch-level quality control was applied, including call-rate thresholds (>98%), sex concordance checks, contamination assessment, and repeat genotyping where necessary. Array data were used for ancestry inference, additional quality control, and concordance checks with matched whole-genome sequencing data. We constructed a global polygenic score for type 2 diabetes to capture overall genetic susceptibility using variants and effect-size weights derived from a previously published genome-wide association study that did not include participants from the present study (25). Genetic variants were filtered to retain only those passing standard quality control criteria (26). Posterior SNP effect sizes were inferred using PRS-continuous shrinkage (PRS-CS), a Bayesian polygenic scoring method that models linkage disequilibrium using continuous shrinkage priors, with the global scaling parameter auto-estimated (27). Polygenic scores were computed using ancestry-specific variants and weights. Genetic ancestry was defined using predicted ancestry assignments provided as auxiliary data by the All of Us Research Program. We assigned participants to genetically inferred ancestry groups based on principal component analysis (PCA) comparisons with a population genetic reference panel, restricting analyses to individuals with an ancestry probability exceeding 0.7. Within each ancestry group, individuals were further excluded if their values on the first two principal components of the genomic relationship matrix were more than four standard deviations from the group centroid.

### Ascertainment of type 2 diabetes

Incident type diabetes cases were identified using any incident billing code in EHR. We excluded any new diagnoses coded during the first six months of monitoring, assuming that such conditions were likely prevalent but not yet recognized clinically. The EHR data from different participating sites were mapped and harmonized using the Observational Medical Outcomes Partnership common data model (28). In Inter99, we used data from separate nationwide health registries to define type 2 diabetes events.

### Statistical analysis

A flow diagram describes how many participants were excluded based on the criteria used to create the analytical dataset (Fig. S1 (21)). Descriptive statistics for participant's demographic and clinical characteristics were presented by median and IQR for continuous variables and frequency for categorical variables.

We used Cox proportional hazard models to examine associations of genetic risk and step counts with type 2 diabetes risk. For these analyses, we censored cases that occurred 6 months after completing enrollment to mitigate potential bias due to time-varying confounding. Person-time for each participant was calculated from the six months after completing baseline assessment to the diagnosis of type 2 diabetes, death, loss to follow-up, or the end of the follow-up period, whichever came first. We modeled polygenic scores and step counts as continuous variables. We averaged daily step counts within each month and subsequently treated step counts as a time-varying covariate in the Cox models to account for month-to-month variations in physical activity during the follow-up period. We also classified participants according to categories of genetic risk and physical activity (nine categories based on thirds of genetic risk and step counts, with low genetic risk and high physical activity as reference) and conducted stratified analyses. We adjusted the multivariable models for age (in years, continuous), sex (male, female), and genetic ancestry inferred from the first 10 PCA of the genomic relationship matrix. We verified the proportional hazards assumption of the Cox model by using the Schoenfeld residuals technique (*P* values for the global test > .1). Analyses were conducted for complete cases, given the small number of people with missing covariates. In secondary analyses, we further adjusted our models for BMI (quintiles of kg/m^2^). BMI was not included in the primary models because it may act as either a mediator or a collider in the association between physical activity and T2D risk (29).

We tested for additive and multiplicative interactions of genetic risk and physical activity with type 2 diabetes incidence. For additive interactions, we assessed the relative excess risk due to interaction (RERI) and further examined the decomposition of the joint effect—that is, the population attributable proportions to genetic risk alone, physical activity alone, and to their interaction (30). For these analyses, we used the following formula (RERI = RR11—RR10—RR01 + 1) and (attributable proportion (AP) = RERI/RR11) (30). CIs for each of the interaction measures were calculated using the delta method described by Hosmer and Lemeshow. We tested for multiplicative interactions using the log-likelihood ratio test to compare the goodness of fit of a multivariable-adjusted model with and without the cross-product interaction term (31). For these analyses, we considered genetic risk and physical activity as continuous variables and modeled the effect per 1SD.

In secondary interaction analyses, we conducted further adjustments for alcohol intake (yes/no), smoking status (ever/never), and socioeconomic factors, including income and educational attainment (college vs some/no college). We also conducted a separate analysis using race/ethnicity instead of ancestry. We further derived device-based measures of physical activity intensity, including daily lightly active minutes, fairly active minutes, and very active minutes. These intensity levels were defined using metabolic equivalents (METs): 1.5-3.0 METs for lightly active, 3.0-6.0 METs for fairly active, and >6.0 METs for very active minutes (32, 33). To further validate these findings, we also incorporated total self-reported physical activity data from 6784 participants from the Inter99 study (18), followed during a median follow up of 20 years, categorizing participants as having high (7-12 hours/week) or low (0-2 hours/week) activity. Additional analyses were conducted stratifying participants by ethnic group (European, African, East Asian, and South Asian) and by sex. To address potential selection bias due to differences between included and non-included participants in age, sex, income level, and ethnicity, we conducted a sensitivity analysis using inverse probability weighting.

Two-sided tests were used to assess whether polygenic risk for type 2 diabetes and physical activity is associated with type 2 diabetes incidence. A one-sided test was used to assess whether RERI is greater than 0, ie, testing the hypothesis that the combined effects of polygenic risk for type 2 diabetes and physical activity exceed their individual effects. All statistical analyses were performed using R software, version 4.0.3 (R Foundation). We followed the Better Precision-data Reporting of Evidence from Clinical Intervention Studies & Epidemiology (BePRECISE) reporting guideline (34).

## Results

Of 14 200 participants with available Fitbit data at the time of our analysis, 9501 had valid physical activity data, genetic information, and no diabetes at baseline (Fig. S1 (21)). The median age was 56 years (IQR 42-66), the median BMI was 28.3 kg/m^2^ (IQR 24.8-33.3), and most participants were women (72%), of European ancestry (88%), and held a college degree (72%; Table 1). Participants contributed 11.6 million person-days of Fitbit monitoring over a median of 5.7 years (IQR, 3.6-7.7), yielding 97.9 billion recorded steps. Median daily step count was 7891 (IQR, 5748–10 471), and step count showed a normal distribution (Fig. S1 (21)). Compared to non-included All of US participants, included individuals were more likely to be younger, female, of European ancestry, with lower BMI and higher educational attainment (Table S1 (21)).

**Table 1:** Baseline characteristics of the included All of US research program participants

	All	Low PRS tertile	Medium PRS Tertile	High PRS Tertile
Person-y^ϕ^	53 672	18 180	17 991	17 501
Age, years	56 (42-66)	57 (43-67)	56 (41.5-67)	55 (41-66)
Sex, Women, No. (%)	6863 (72)	2287 (72%)	2265 (72%)	2311 (73%)
BMI, kg/m^2^	28.3 (24.8-33.3)	27.3 (24.0-31.8)	28.3 (24.8-33.2)	29.4 (25.7-35.0)
Smoker, No. (%)	3136 (33)	1014 (32%)	1067 (34%)	1055 (33%)
Alcohol User, No. (%)		3069 (97%)	3088 (98%)	3073 (97%)
Self-reported race/ethnicity^ε^				
White, No. (%)	8132 (86)	2862 (90%)	2847 (90%)	2835 (90%)
African, No. (%)	583 (6)	205 (6%)	219 (7%)	227 (7%)
East Asian, No. (%)	207 (2)	69 (2%)	74 (2%)	73 (2%)
South Asian, No. (%)	85 (1)	31 (1%)	27 (1%)	32 (1%)
Education				
College degree, No. (%)	6856 (72)	2334 (74%)	2291 (72%)	2231 (70%)
Some/No college, No. (%)	2587 (27)	819 (26%)	850 (27%)	918 (29%)

Abbreviation: PRS, polygenic risk score.

Values are medians (interquartile range) for continuous variables; numbers and (percentages) for categorical variables.

During 53 672 person-years of follow-up, 494 incident cases of type 2 diabetes were documented. Crude type 2 diabetes incidence rates per 1000 person-year according to daily step counts tertiles were 13.5 (95% CI: 11.9-15.2), 7.5 (95% CI: 6.3-8.8), and 5.9 (95% CI: 4.7-7.0) for the lowest, intermediate, and highest tertile, respectively (multivariable-adjusted HR of 0.40 (95% CI: 0.26, 0.63; Table 2). Each standard deviation (SD) increase in step count was associated with lower risk of type 2 diabetes, with an adjusted HR of 0.65 (95% CI, 0.57-0.73). Compared with women, men had a significantly higher risk of developing T2D, with a multivariable adjusted HR of 1.62 (95% CI: 1.52-1.72). Men also had a higher number of daily step count, with an estimated median of 8726 steps/day compared with 7767 steps/day in women, corresponding to a difference of 959 steps/day (95% CI: 818-1100; *P* < .001; Fig. S2 (21)).

**Table 2:** Adjusted hazard ratios of type 2 diabetes risk according to tertiles of daily step counts and genetic risk

	Low step count	Intermediate step count	High step count	*P* for trend
No. of events/person-years	263/19 415	136/18 046	95/16 210	—
Incidence rate (1000 person-years; 95% CI)	13.5 (11.9-15.2)	7.5 (6.3-8.8)	5.9 (4.7-7.0)	—
Multivariable-adjusted model	1.00 (Ref)	0.68 (0.57-0.82)	0.40 (0.26-0.63)	<.001

	Low genetic risk	Intermediate genetic risk	High genetic risk	*P* for trend
No. of events/person-years	107/18 180	142/17 991	245/17 501	—
Incidence rate (1000 person-years; 95% CI)	5.9 (4.8-7)	7.9 (6.6-9.2)	14 (12.2-15.8)	—
Multivariable-adjusted model	1.00 (Ref)	1.59 (1.43-1.76)	2.50 (2.04-3.06)	<.001

Hazards ratios and 95% CI for type 2 diabetes risk. Daily step counts and genetic risk were categorized in three categories based on the distribution of physical activity and polygenic score. Cox proportional hazards models were adjusted for age (in years, continuous), sex (male, female), and the first 10 principal components of the genomic relationship matrix

Similarly, type 2 diabetes incidence increased across tertiles of polygenic risk, with incidence rates per 1000 person-years of 5.9 (95% CI: 4.8, 7.0) in the lowest, 7.9 (95% CI: 6.6, 9.2) in the intermediate, and 14.0 (95% CI: 12.3, 15.8) in the highest tertile. In adjusted models, participants in the highest tertile had 2.50 times the risk of type 2 diabetes compared with those in the lowest (95% CI, 2.04-3.06; Table 2). When modeled in the continuous scale, each SD increase in polygenic score was associated with an adjusted HR of 1.52 (95% CI, 1.38-1.67).

**Figure 2:**
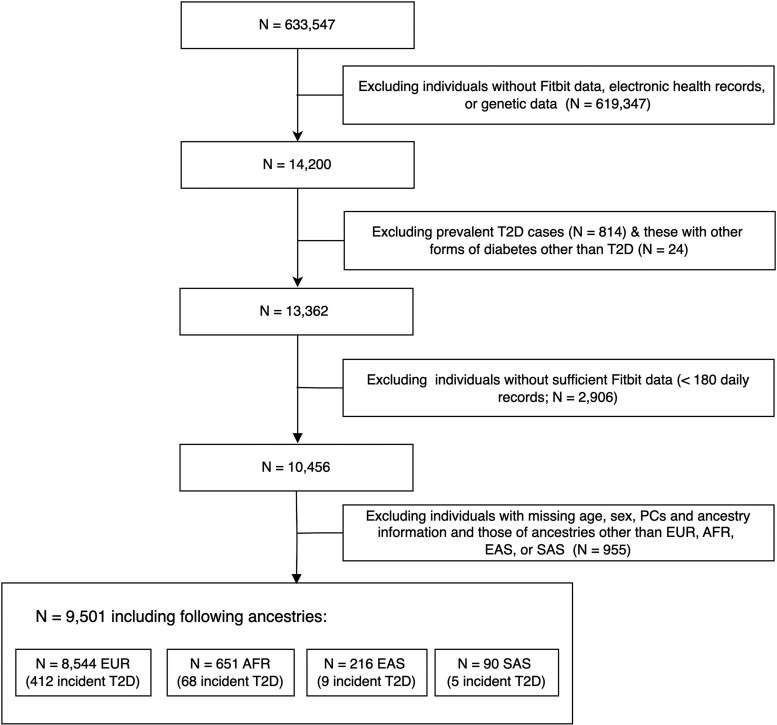
Risk of incident type 2 diabetes by global polygenic risk score for type 2 diabetes, physical activity. Risk is indexed using crude incidence rate and hazard ratio adjusted for age, sex, and genetic ancestry. HR hazard ratio. Dots correspond to averages of daily step count tertiles, which are ∼ 5k, ∼ 8k, and ∼13k. Histograms along the y-axis (to the right) show the density of daily step counts. Incidence rates are computed for tertiles of daily step counts.

When examining joint associations, crude incidence rates ranged from 4.1 (95% CI, 2.5-5.7) per 1000 person-years among participants with low genetic risk and high physical activity to 18.4 (95% CI, 15.2-21.6) among those with high genetic risk and low physical activity (Fig. 2). The corresponding multivariable-adjusted HR was 6.20 (95% CI, 3.97-9.60). Within the high genetic risk group, individuals with low activity (∼5000 steps/day) had nearly double the incidence of those with high activity (∼13 000 steps/day; HR, 1.92; 95% CI, 1.36-2.69). The joint association was higher than the sum of the risk associated with each factor alone, with a relative excess risk due to interaction (RERI) of 0.20 (95% CI: 0.04, 0.36); *P* = .007). Decomposition of excess type 2 diabetes risk attributed 40% to genetic risk (95% CI: 31, 49), 45% to low physical activity (95% CI: 29, 62), and 15% to their interaction (95% CI: 3, 27; Table 3). No evidence of multiplicative interaction was observed (*P* > .05).

**Table 3:** Additive interactions between physical activity and global polygenic risk of type 2 diabetes

**Main effects**	
Physical activity*^a^*	1.53 (1.32, 1.74)
Polygenic score*^b^*	1.61 (1.32, 1.74)
Joint effect	2.34 (1.87, 2.81)
**Relative excess risk due to interaction**	
Relative excess risk due to interaction	0.2 (0.04, 0.36)
*P* value	0.007
**Attributable risk proportion, %**	
Daily step count	39.8 (31.0, 48.5)
Polygenic score	45.4 (28.6, 62.1)
Additive interaction	14.8 (2.5, 27.2)

Multivariable adjusted risk of type 2 diabetes estimated from Cox proportional hazards models adjusted for age, sex, and the first 10 principal components of the genomic relationship matrix. The joint analysis was conducted to quantitatively estimate the combined association of physical activity and genetic risk simultaneously with the incidence of type 2 diabetes. The relative excess risk due to interaction (RERI) is based on joint effects of a one-standard-deviation increase in PRS and a one-standard-deviation decrease in daily step count using the following formula (RERI = RR11—RR10—RR01 + 1). The model's attributable risk proportion due to the interaction was calculated as AP = RERI/RR11(28)

^*a*^per SD decrease in daily step counts.

^*b*^per SD increase in the global polygenic scores.

We conducted a series of sensitivity and subgroup analyses. When physical activity was modeled by intensity, similar additive interactions were observed. The RERI was 0.21 (95% CI, 0.02-0.41; *P* = .015) for moderate activity minutes, with 19% of excess risk attributable to interaction, and 0.40 (95% CI, 0.21-0.59; *P* < .001) for highly active minutes, with 31% attributable to interaction (Table S2 (21)). Adjustment for BMI did not materially change these estimates (Table S2 (21)), and additional adjustment for alcohol intake, smoking status, and socioeconomic factors likewise had no meaningful impact on the results (Table S3 (21)). Similar findings were observed when race/ethnicity was included in the models instead of ancestry (Table S4 (21)).

In the Inter99 cohort, which had a younger age distribution, lower mean BMI, and a higher proportion of females and participants from European ancestry compared with the All of Us cohort (Table S1 (21)), 371 incident cases occurred over 103 916 person-years of follow-up. Crude incidence rates ranged from 1.6 per 1000 person-years (95% CI: 0.4-2.8) among participants with low genetic risk and high physical activity to 11.0 per 1000 person-years (95% CI: 7.8-14.2) among those with high genetic risk and low physical activity (Fig. S3 (21)). The RERI was 0.53 (95% CI: 0.30-0.76; *P* = .007), with a 44% of the excess risk attributable to the interaction between genetic risk and physical inactivity (Table S5 (21)). Adjustment for BMI in Inter99 attenuated this proportion to 38% (95% CI, −6 to 82; Table S5 (21)). In ancestry-stratified analyses, no significant associations between genetics and physical activity or interactions were observed in the African ancestry group (68 cases among 583 participants; Table S6 (21)). Due to small sample sizes, models for South Asian (5 cases among 85 participants) and East Asian (9 cases among 207 participants) did not converge. Sex-stratified analyses showed additive interactions in both sexes. Among men, the RERI was 0.2 (95% CI: 0.01, 0.42; *P* = .03), and 22% of the excess risk was attributed to interaction. Among women, RERI was 0.2 (95% CI: −0.04, 0.43; *P* = .050), with an attributable proportion of 11% (Table S7 (21)). Finally, reweighting the sample to address potential selection bias due to differences between included and non-included participants in age, sex, income level, and ethnicity, did not alter the results, and the additive interaction between physical activity and genetic susceptibility on the risk of T2D remained statistically significant (RERI = 0.21, 95% CI: 0.05, 0.37, *P* = .005; Table S8 (21)).

## Discussion

Our findings provide consistent evidence of an additive interaction between genetic risk and physical activity in the development of type 2 diabetes. Specifically, individuals with both high genetic risk and low step counts had a higher risk than expected from the sum of their individual effects, with approximately one-fifth of the excess risk attributable to this interaction. Similar patterns were observed when physical activity was assessed using device-based intensity metrics or self-reported total physical activity. Since additive interactions reflect differences in absolute risk across populations (30), these observations may have potential public health relevance. Specifically, reducing the co-occurrence of high genetic susceptibility with low levels of physical activity could contribute to lowering type 2 diabetes incidence, supporting both targeted lifestyle interventions for high-risk individuals and population-level strategies that promote physical activity to mitigate inherited risk. However, replication in other cohorts and settings will be important to better understand the robustness and generalizability of these results.

Our study builds on prior research investigating gene–environment interactions in type 2 diabetes, which has often yielded inconsistent findings (10-13). One likely contributor to these discrepancies is error in exposure assessment due to reliance on self-reported methods (14, 15). Using accelerometer-based physical activity measures, a recent UK Biobank study demonstrated an additive effect between physical activity and polygenic scores on type 2 diabetes risk, with greater absolute risk reductions observed among individuals at high genetic risk who engaged in moderate-to-vigorous physical activity (19). Our findings extend this evidence base in three important ways. First, unlike the UK Biobank study, which relied on a single time-point physical activity assessment, we modeled physical activity as a time-varying exposure, capturing behavioral changes over a median follow-up of 5 years. This approach more accurately reflects behavioral dynamics leading up to diabetes onset (35). Second, we used daily step counts as the primary exposure. Compared with intensity-based classifications such as “moderate-to-vigorous,” which may vary in interpretation and are less intuitive, step counts provide a simple, interpretable, and actionable metric for public health messaging (16). Third, our polygenic scores were derived from an external discovery dataset that excluded participants from this study, thereby mitigating potential inflation of type I error due to sample overlap, a methodological limitation that may have influenced the UK Biobank analyses. Together, our study strengthens the evidence that objectively measured physical activity interacts with genetic risk on the development of type 2 diabetes.

Physical activity remains central to public health recommendations for type 2 diabetes prevention (36), yet current guidelines do not account for heterogeneity in individual responses. Factors such as genetic susceptibility, body composition, and diet can all influence the effectiveness of physical activity (37). For example, in the ARIC cohort, the protective effects of physical activity were attenuated in women with higher genetic predisposition to insulin resistance (38), suggesting that greater physical activity may be needed to offset genetic risk. Similarly, a prior analysis from the All of Us Research Program estimated that individuals at high genetic risk for obesity would need to walk an additional ∼2300 steps per day to match the obesity risk of those at lower risk (39). In our study, participants at high genetic risk who walked ∼5000 steps per day had nearly double the incidence of type 2 diabetes compared to those walking ∼13 000 steps per day. Our models estimated that 15% of type 2 diabetes cases could have been prevented by eliminating one of the two risk factors, emphasizing the potential impact of increasing activity levels, particularly among genetically susceptible individuals.

Although the proportion of risk attributable to gene–physical activity interactions was similar when using step counts or device-based intensity measures, higher estimates were observed in the Inter99 cohort for total physical activity. This discrepancy may reflect differences in follow-up time, population characteristics such as age and obesity prevalence, or potential inflation due to self-reported activity measures. Since population-attributable risk depends on both exposure prevalence and effect size (40), exposure misclassification, especially with self-reported physical activity, could partly explain these differences. While these excess risk estimates due to the interaction may change over time with shifting diabetes incidence, our findings underscore the ongoing relevance of gene–physical activity interactions in type 2 diabetes.

This study has several limitations. First, as an observational study, participants were not randomized to high- or low-physical activity groups, limiting causal inference interpretations. Recall-by-genotype studies could help test these hypotheses under experimental conditions. Second, the Wearables Enhancing All of Us Research (WEAR) initiative provided Fitbit devices to participants from communities underrepresented in medical research, helping to reduce barriers related to device access. However, despite this effort and our use of inverse probability weighting to address potential selection bias arising from differences between included and non-included participants in age, sex, income level, and ethnicity, some degree of selection bias may still remain. Third, incident type 2 diabetes was defined solely based on any incident billing codes from the electronic health record, which may introduce misclassification. However, this approach is widely used in biobank research, including other All of Us analyses (17, 41). Fourth, the number of type 2 diabetes cases was relatively small, reflecting limited follow-up and the subset of participants with linked wearable data. Fifth, baseline fasting glucose and HbA1c were available for only a subset of participants at the time of analysis. Sixth, while we used comprehensive polygenic scores for type 2 diabetes, other genetic risk factors (ie, for obesity) were not included. Although we adjusted for BMI in secondary analyses, residual confounding, collider bias, or mediation effects could still exist. Larger studies with longer follow-up and detailed modeling of BMI or weight gain changes during the observed period may help address this. Finally, other molecular, clinical, and behavioral factors such as diet, body composition, and the gut microbiome were not included in our analyses because these data were not available. In particular, the lack of detailed dietary information and dietary pattern data represents an important limitation of this study and should be considered when interpreting our findings.

In conclusion, by quantifying the combined contributions of genetic risk and physical activity to type 2 diabetes risk, our results underscore the potential value of integrating genomic data with objective, device-derived measures of physical activity to better identify those who are most likely to benefit from targeted interventions. Future studies with larger, diverse populations and longer follow-up are needed to confirm these interactions and to evaluate how they can be translated into effective public health and clinical interventions.

## Data Availability

The datasets used for this study are freely available via the NIH All of Us Researcher Workbench (https://www.researchallofus.org/data-tools/workbench/) to approved researchers with institutional access. The relevant analytical code used for this analysis is available on the Researcher Workbench or from the leading author (Dr. Xuan Zhou: xuan.zhou@sund.ku.dk) upon request. The genotype and phenotype data from Inter99 used in this study are not publicly available but are available from the corresponding authors on reasonable request. The Inter99 data sets may be obtained by a third party by contacting Allan Linneberg at allan.linneberg@regionh.dk.
